# Resistance Exercise Intervention Restores Functional Capacity and Improves Frailty Biomarkers in Centenarians

**DOI:** 10.1002/jcsm.70079

**Published:** 2025-10-02

**Authors:** Diego Marcos‐Perez, Adrián Hernandez‐Vicente, Sara Cruces‐Salguero, Jon Landa, Michelle Bonvini, German Vicente‐Rodríguez, Esther Pueyo, Leocadio Rodriguez‐Mañas, Pedro Abizanda, David Otaegui, Nuria Garatachea, Ander Matheu

**Affiliations:** ^1^ Cellular Oncology Group, Biodonostia (Biogipuzkoa) Health Research Institute San Sebastián Spain; ^2^ Growth, Exercise, Nutrition and Development (GENUD) Research Group University of Zaragoza Zaragoza Spain; ^3^ Faculty of Health and Sport Science University of Zaragoza Huesca Spain; ^4^ Red Española de Investigación en Ejercicio Físico y Salud en Poblaciones Especiales (EXERNET) Zaragoza Spain; ^5^ BSICoS Group, I3A, IIS Aragón Universidad de Zaragoza Zaragoza Spain; ^6^ Centro de Investigación Biomédica en Red de Bioingeniería, Biomateriales y Nanomedicina Madrid Spain; ^7^ Geriatrics Department Getafe University Hospital Getafe Spain; ^8^ Centro de Investigación Biomédica en Red Fragilidad y Envejecimiento Saludable (CIBERFES) Madrid Spain; ^9^ Geriatrics Department Complejo Hospitalario Universitario de Albacete Albacete Spain; ^10^ Facultad de Medicina de Albacete Universidad de Castilla‐La Mancha Albacete Spain; ^11^ Neuroimmunology Group, Biodonostia (Biogipuzkoa) Health Research Institute San Sebastián Spain; ^12^ Neurodegenerative Diseases Research Area of CIBER (CIBERNED) Madrid Spain; ^13^ Faculty of Health and Sport Sciences University of Zaragoza Huesca Spain; ^14^ Instituto Agroalimentario de Aragon‐IA2‐CITA Zaragoza Spain; ^15^ Centro de Investigación Biomédica en Red de Fisiopatología de la Obesidad y Nutrición (CIBER‐Obn) Madrid Spain; ^16^ IKERBASQUE, Basque Foundation for Science Bilbao Spain

**Keywords:** centenarians, frailty, molecular biomarkers, resistance exercise

## Abstract

**Background:**

Centenarians comprise an age group characterized by exceptional longevity and low age‐associated pathologies. However, they still experience physiological decline, and different studies have linked frailty to this population. Exercise interventions reverse frailty and improve functional capacity, but no studies have addressed the effect of an intervention in centenarians. In this study, we assessed the impact of a 12‐week resistance exercise intervention in a group of centenarians and characterized their functional capacity as well as the expression of several molecular biomarkers associated with frailty.

**Methods:**

A total of 19 centenarians were enrolled, but 7 of them did not complete the study. The remaining 12 centenarians were randomly assigned to the control or intervention group, which was a 12‐week resistance exercise intervention. Molecular biomarkers were measured by qRT‐PCR and ELISA.

**Results:**

The intervention group improved their functional capacity measured by Short Physical Performance Battery (SPPB) (post 5.0 vs 2.3 in pre) and Physical Performance and Mobility Examination (PPME) (6.5 vs 3.8), as well as in frailty status studied by Fried Frailty Phenotype (3.0 vs 3.8) and Frailty Trait Scale 5 (FTS5) (post 30.7 vs 34.0 in pre) scales. ANCOVA revealed that the resistance training led to significant improvements in functional capacity scales SPPB (*p* = 0.01) and PPME (*p* < 0.001), as well as Fried Frailty Phenotype (*p* = 0.001) and FTS5 (*p* = 0.05). Biomarkers related to frailty *(EGR1*, *miR194‐5p, miR125b‐5p* and *miR454‐3p)* and inflammation (*IL‐*6 and *IL‐1β)* showed different expression patterns in centenarians (*n* = 19) compared to both old (*n* = 44, average of 79 years old) and young adults (*n* = 34, average of 29 years old) groups. Notably, the intervention was associated with improvements in frailty and inflammation biomarkers expression. Finally, correlation analyses showed significant associations between all functional and frailty variables, with SPPB correlating with *miR454‐3p (ρ = 0.73)* and FTS5 correlating with *miR454‐3p (ρ = −0.83), IL‐6 (ρ = 0.60)* and *miR125b‐5p (ρ = −0.55)*.

**Conclusions:**

Our results revealed that resistance exercise intervention enhances functional status and reduces frailty in centenarians, and this is associated with improvements in frailty and inflammation biomarkers.

## 1 Introduction

Centenarians comprise an age group that exhibits extreme longevity. This longevity typically coincides with remarkably low incidence rates of common and lethal age‐associated pathologies, including cancer, stroke, cardiovascular diseases and neurodegenerative disorders. Moreover, a significant percentage of centenarians, despite some cross‐country variability, maintain some level of independence and are able to perform basic activities of daily life [1]. However, centenarians are not free of age‐associated decline in several systems, and different studies have linked this population to frailty. In this sense, Gu et al. [2] and Herr et al. [3] showed that Chinese and European centenarians are more frail than non‐centenarians older adults, thus suggesting that centenarians undergo an exacerbated decline in physiological and functional capacity during the final years of life. Similarly, we have previously shown that centenarians engage in less physical activity than nonagenarians [4].

Frailty represents a dynamic condition that can be reversed or attenuated through targeted interventions, which can comprise different activities, including physical activity (PA), exercise, dietary modifications and/or multicomponent interventions. Among them, exercise is the most well‐established intervention to prevent, ameliorate and reverse the physiological and functional age‐related decline in older adults. Nevertheless, there are no studies to date that assess the impact of an exercise intervention on the functional performance and frailty status of centenarians.

Frailty has traditionally been evaluated using the Fried's phenotype, which focuses on PA, or the Frailty Index (FI), that quantifies the accumulation of age‐related health deficits exhibited by the individual. Aiming to complement these functional tests or scales, different studies have sought to identify potential biomarkers of frailty and uncover, at a biological level, the underlying molecular mechanisms driving its progression [5]. In this regard, most insights into the physiopathology of frailty derive from independent observational studies, which have consistently detected increased inflammation‐related genes or cytokines in blood samples from frail individuals [5, 6]. In particular, *IL‐6* and *IL‐1β* have been extensively correlated with increased frailty levels. Additionally, two independent *omics*‐based studies—one conducted in Europe under the FRAILOMIC Consortium [7] and another in the United States [8]—analysed frail and robust individuals using global, unbiased approaches. Their findings suggest that three miRNAs (*miR125, miR194* and *miR454*) and *EGR1* are differentially expressed in frail individuals. However, no research to date has investigated the expression of these biomarkers in centenarians and their potential reversibility in response to an exercise intervention. Therefore, the aim of this study was to assess the effects of a 12‐week resistance exercise intervention in a centenarian cohort and to determine its impact on functional capacity and frailty as well as in the expression of selected frailty‐associated biomarkers.

## 2 Materials and Methods

### 2.1 Study Population, Exercise Intervention and Functional Scales

**FIGURE 1:**
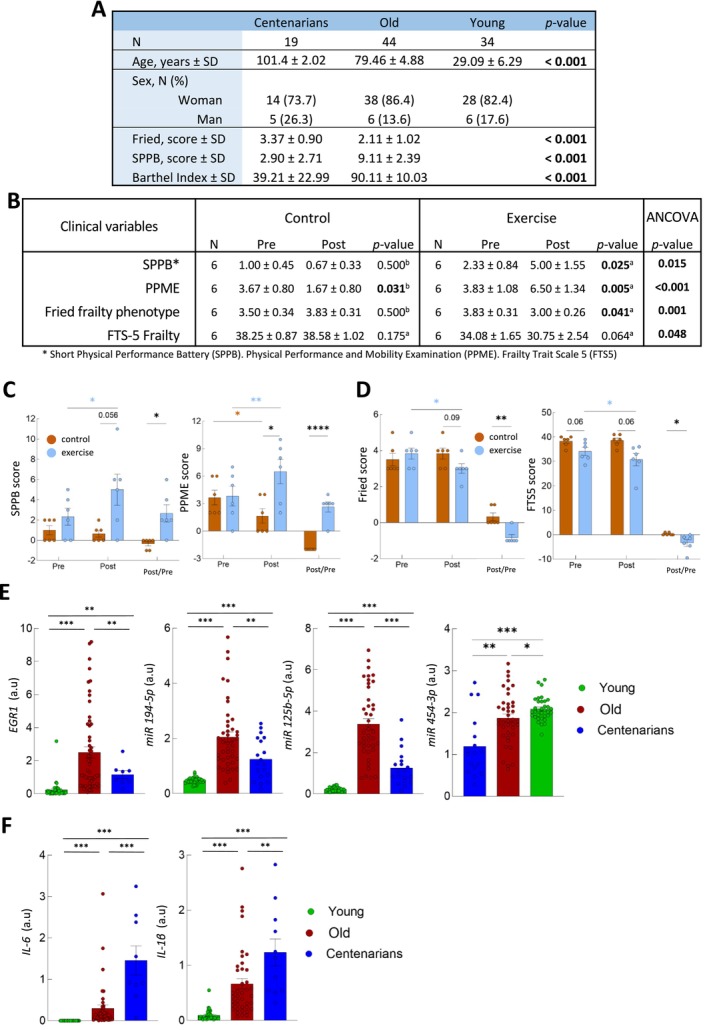
Description of populations of the study and the impact of the intervention (A) Description of centenarians (*n* = 19), old (*n* = 44) and young adults (*n* = 34) populations. (B) Table with functional and frailty scales in control and intervention groups before and after 12‐week resistance exercise intervention. ANCOVA analysis of clinical data. (C, D) Individual graphs and results in functional and frailty scales in control and intervention group pre and post intervention (E, F) mRNA expression of *EGR1*, *miR194‐5p*, *miR125‐5p* and *miR454‐3p* levels in young (*n* = 34) old (*n* = 44) and centenarians (*n* = 19) groups. (F) expression of *IL‐6* and *IL‐1β* in same groups.

Centenarians were institutionalized and evaluated directly at their geriatric nursing home by the same research team, applying consistent procedures and standardized equipment. We enrolled 19 volunteers aged 100 years and older. However, seven individuals did not complete the intervention due to COVID‐19 lockdown. We randomly assigned the remaining centenarians to either control (*n* = 6, women: 6) or intervention group (*n* = 6, women: 4). The control group received usual care, while the intervention group participated in supervised resistance training twice a week over a 12‐week period. Each session included 8 exercises performed in 1 to 3 sets of 8 to 10 repetitions, at intensities ranging from 50% to 70% of the estimated one‐repetition maximum. Training load and the number of sets were adjusted biweekly based on participants' evolving physical capacity. We measured functional and frailty status at baseline and after the 12‐week programme, including Short Physical Performance Battery (SPPB), Physical Performance and Mobility Examination (PPME) [9], Fried Frailty Phenotype and Frailty Trait Scale 5 (FTS5) [10]. The team collected blood samples from centenarians both preintervention and postintervention. To provide comparative data, we also included blood samples from two additional groups: 44 old individuals (mean age: 79.5 years) and 34 young individuals (mean age: 29.1 years) (Figure 1A).

### 2.2 Ethics

This study was approved by the ethical committee for clinical research of Aragón, Spain (#PI18/381) and CEIm‐E of Euskadi (PI2021023) and adhered to the tenets of the Declaration of Helsinki.

### 2.3 RNA Extraction and Quantification

We extracted total RNA from whole blood samples using QIAamp RNA Blood Mini Kit (Qiagen) and Maxwell RSC miRNA Plasma and Serum Kit (Promega), respectively. We then measured mRNA and miRNA by quantitative real‐time polymerase chain reaction (qRT‐PCR). We calculated relative quantification using the 2^−ΔΔCt^ formula. mRNA expression was normalized to glyceraldehyde 3‐phosphate dehydrogenase (*GAPDH*) and miRNA expression to *miR191‐5p*.

### 2.4 Statistical Analysis

We reported results as mean ± standard error of the mean (SEM). We tested data normality using the Shapiro–Wilk test and compared paired samples with both Student's t‐test and the Wilcoxon test. To evaluate differences across age groups, we performed one‐way ANOVA followed by multiple comparisons, applying the false discovery rate (FDR) method to correct for multiple testing. We assessed the intervention's effect on clinical variables using analysis of covariance (ANCOVA). To identify associations between biomarkers and clinical variables, we calculated Spearman's rank correlations. Statistical significance was as follows: #, *p* ≤ 0.1, *p* ≤ 0.05 *, *p* ≤ 0.01 ** and *p* ≤ 0.001 ***.

## 3 Results and Discussion

Previous research suggests that regular exercise positively enhances health and independence in centenarians [11]. However, no studies to date have evaluated how exercise interventions affect their functional capacity and frailty status. To address this issue, we characterized a cohort of centenarians and randomly assigned 12 centenarians into control and intervention groups (*n* = 6 each). Noteworthy, the intervention group showed improvements in SPPB (post 5.0 vs. 2.3 in pre), PPME (6.5 vs. 3.8), Fried (3.0 vs. 3.8) and FTS5 (post 30.7 vs. 34.0 in pre) scales (Figure 1B–D). In contrast, the control group only showed modest or no improvements in these scales (Figure 1B–D). In line with these differences, the training effect (control vs. intervention changes) measured by ANCOVA was statistically significant for all functional and frailty measurements (Figure 1B). These results reveal that personalized resistance exercise intervention enhances both functional capacity and frailty status in centenarians.

Next, molecular studies were performed. First, we compared the expression of a set of molecular biomarkers linked to frailty in blood samples from centenarians, old and young individuals. Among the genes and miRNAs previously linked to frailty, we found that *EGR1*, *miR194‐5p* and *miR125b‐5p* presented higher mRNA levels in old vs. young individuals (Figure 1E). Moreover, centenarians displayed reduced expression compared to old individuals, reaching levels similar to those of young individuals (Figure 1E). This similar expression pattern between centenarians and young individuals has been previously observed for both mRNA and miRNAs [12, 13]. *miR454‐3p* showed lower levels in centenarians compared to old and young individuals (Figure 1E). On the other hand, the inflammatory markers *IL‐6* and *IL‐1β* displayed significantly increased mRNA levels with age (Figure 1F). These results are consistent with the concept of ‘inflammaging’, a condition characterized by low‐grade, chronic and systemic upregulation of the inflammatory response that intensifies with age and from which centenarians are not exempt. Indeed, several studies have reported higher levels of *IL‐6* and *IL‐1β* [14] in centenarians compared to both old and young individuals.

**FIGURE 2:**
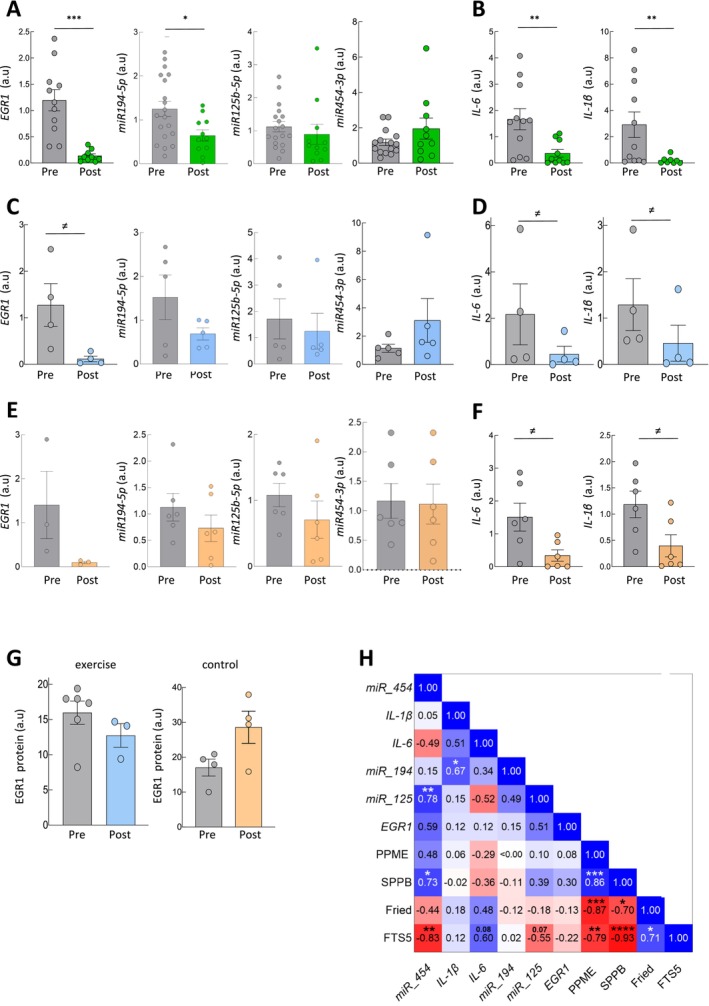
Frailty biomarkers in intervention. (A, B) Expression levels of markers in entire pre/post centenarian cohort (*n* = 19). (C, D) mRNA expression of indicated biomarkers in pre and post exercise in intervention group (*n* = 6). (E, F) mRNA expression levels of markers in pre and post condition in control group (*n* = 6). (G) EGR1 protein expression by ELISA in pre/post samples of control and exercise group (*n* = 6 each). (H) Spearman's correlations between clinical variables and markers expression.

Next, we measured the expression levels of selected genes both before and after the intervention. Among the frailty biomarkers, *EGR1*, *miR194‐5p* and *miR125b‐5p* levels declined (fold change of 0.2, 0.6 and 0.7, respectively), although only *EGR1* and *miR194‐5p* showed statistically significant reductions in the postintervention sample (Figure 2A). In the same line, the inflammation biomarkers *IL‐*6 and *IL‐1β* decreased significantly (fold change 0.35 and 0.5, respectively) after the intervention (Figure 2B). In contrast, *miR454‐3p* was higher with the intervention (Figure 2A). These results indicate that the intervention attenuated the expression of several biological markers associated with frailty and inflammation. It should be taken into account that previous results described higher levels of *EGR1, miR194‐5p*, *IL‐*6 and *IL‐1β* and lower levels of *miR454‐3p* and *miR125b‐5p* in frail individuals compared with robust individuals [7, 8].

Next, we characterized the effect of the supervised resistance training of a 12‐week period twice a week on the genes of interest. At a molecular level, the exercise group exhibited posttraining declines in mRNA levels of all frailty and inflammation biomarkers, except *miR454‐3p* (Figure 2C,D). Even if these reductions did not reach statistical significance, a decreasing trend could be observed in EGR1, *IL‐6* and *IL‐1β (*#, *p* ≤ 0.1). Similar results were observed in the control group, especially in the case of inflammation markers (Figure 2E,F). In order to strengthen the molecular results, we measured EGR1 protein levels by ELISA. In this case, we observed a reduction in EGR1 levels in the exercise group and no differences or increase in the control group (Figure 2G). These findings indicate that there is a reversion in mRNA/miRNA expression in this centenarian cohort for their participation in the study, which is enhanced with the resistance exercise training in the cases of *EGR1*, *miR454‐3p*, *IL‐6* and *IL‐1β*. These results might suggest that the alteration in molecular marker expression precedes physical improvement. The low number of cases per group and the rate of missing mRNA values should be considered when interpreting the limitations of the statistical analysis.

To identify associations between biomarkers and clinical variables, we conducted Spearman's correlation analyses across the entire pre/post intervention cohort. The results revealed strong and significant correlation among all functional and frailty measurements, such as PPME and SPPB (*ρ* = 0.86, *p* < 0.001), Fried and PPME (*ρ* = −0.87, *p* < 0.001), SPPB and both Fried (*ρ* = −0.70, *p* = 0.018) and FTS5 (*ρ* = −0.93, *p* < 0.0001) and Fried and FTS5 (*ρ* = 0.71, *p* = 0.014) (Figure 2H). Besides, *miR194* and *IL‐1β* (*ρ* = 0.67, *p* = 0.035), *miR454* and *miRNA125* (*ρ* = 0.78, *p* = 0.008) showed a positive and significant correlation between the molecular biomarkers. Finally, frailty FTS5 scale correlated significantly with *miR454* (*ρ* = −0.83, *p* = 0.007), *IL‐6* (*ρ* = 0.60, *p* = 0.08) and *miR125* (*ρ* = −0.55, *p* = 0.076) and SPPB with *miR454* (*ρ* = 0.73, p = 0.03) (Figure 2H). These results show a positive correlation between the exercise training and functional activity improvements and molecular biomarkers, which is especially robust with the former.

Overall, our results revealed that the resistance exercise training improved functional capacity and reduced frailty status in centenarians. Moreover, we identified that frailty‐ and inflammation‐associated biomarker expression varied between centenarians and older and younger individuals, with two distinct patterns emerging across age groups. Notably, the resistance exercise intervention was associated with improvements in frailty and inflammation biomarkers in the centenarians.

## Sponsor Role

None.
